# *Bacteroides plebeius* improves muscle wasting in chronic kidney disease by modulating the gut‐renal muscle axis

**DOI:** 10.1111/jcmm.17626

**Published:** 2022-12-02

**Authors:** Tingting Pei, Daoqi Zhu, Sixia Yang, Rong Hu, Fujing Wang, Jiaxing Zhang, Shihua Yan, Liliang Ju, Zhuoen He, Zhongxiao Han, Jinyue He, Yangtian Yan, Mingqing Wang, Wei Xiao, Yun Ma

**Affiliations:** ^1^ Department of Traditional Chinese Medicine Southern Medical University Guangzhou Guangdong China; ^2^ Key Laboratory of Glucolipid Metabolic Disorder, Ministry of Education Guangdong Pharmaceutical University Guangzhou Guangdong China; ^3^ Department of Pharmacy Nanfang Hospital, Southern Medical University Guangzhou Guangdong China

**Keywords:** *Bacteroides plebeius*, chronic kidney disease, intestinal microecology, muscle wasting

## Abstract

Chronic kidney disease (CKD) affects approximately 10% of the global population. Muscle atrophy occurs in patients with almost all types of CKD, and the gut microbiome is closely related to protein consumption during chronic renal failure (CRF). This study investigated the effects of *Bacteroides plebeius* on protein energy consumption in rats with CKD, and our results suggest that *Bacteroides plebeius* may combat muscle atrophy through the Mystn/ActRIIB/SMAD2 pathway. A total of 5/6 Nx rats were used as a model of muscle wasting in CKD. The rats with muscle wasting were administered *Bacteroides plebeius* (2 × 10^8^ cfu/0.2 ml) for 8 weeks. The results showed that *Bacteroides plebeius* administration significantly inhibited muscle wasting in CKD. High‐throughput 16 S rRNA pyrosequencing revealed that supplementation with *Bacteroides plebeius* rescued disturbances in the gut microbiota. *Bacteroides plebeius* could also enhance the barrier function of the intestinal mucosa. *Bacteroides plebeius* may modulate the gut microbiome and reduce protein consumption by increasing the abundance of probiotics and reducing damage to the intestinal mucosal barrier. Our findings suggest that *Bacteroides plebeius* may combat muscle atrophy through the Mystn/ActRIIB/SMAD2 pathway.

## 1 INTRODUCTION

Chronic kidney disease (CKD) is a growing public health problem with an estimated prevalence between 8% and 16% worldwide,^1^ and its complications include loss of muscle protein.^2^ Muscle wasting occurs in almost every type of CKD and is an independent risk factor for CKD‐induced morbidity and mortality.^3^ The treatment of muscle wasting results in a greater socio‐economic burden. There is an urgent need to explore the underlying mechanisms and identify more effective treatments for muscle wasting in CKD.

Muscle atrophy is defined as a decrease in the skeletal muscle mass and is an important indicator of the prognosis of CKD.^4^ Protein‐energy wasting (malnutrition) is characterized by abnormal reductions in the body's protein mass and energy reserves and skeletal muscle atrophy, a hallmark of protein consumption^5^ and is prevalent in patients with CKD.^6^ Muscle atrophy in CKD is a complex process caused by impaired insulin‐like growth factor‐1 (IGF‐1)/insulin signalling, metabolic stress, elevated glucocorticoid levels and inflammation.^7^ Insulin resistance is a recognized risk factor of CKD‐related disease,^8^ and inflammation is reportedly a key biological event leading to muscle atrophy.^9^ Studies have also shown that many pro‐inflammatory cytokines, such as tumour necrosis factor‐α (TNF‐α), interleukin‐1 (IL‐1), interleukin‐6 (IL‐6), IL‐1 IL‐6 and TNF‐α, can promote proteolysis in muscles.^10^ Through NF‐κB, a key transcription factor, inflammation promotes the expression of the ubiquitin E3 ligase MuRF‐1 to increase muscle proteolysis via the ubiquitin–proteasome system.^11^ In the cytoplasm, NF‐κB combines with its repressor IκB to form an inactive complex.^12^ NF‐κB is released into the nucleus, where it can promote the expression of specific genes (such as MuRF‐1).^13^ The activation of myostatin/activin signalling is critical for triggering accelerated muscle catabolism, which contributes to muscle loss in a variety of disease states.^14^ The binding of myostatin and activin to the ActRIIB receptor complex on the muscle cell membrane results in the activation of Smad2/3‐mediated transcription,^15^ which in turn stimulates FoxO‐dependent transcription and enhances muscle proteolysis through the ubiquitin–proteasome system and autophagy.^16^ Furthermore, Smad activation inhibits muscle protein synthesis by inhibiting Akt signalling.^17^ Pharmacological blockade of the myostatin/activin‐ActRIIB pathway prevents or reverses the loss of muscle mass and strength in various disease models, including models of cancer cachexia and renal failure.^18^ In addition, this approach can significantly extend the lifespan of animals with cancer‐related muscle loss. Inhibiting the effects of myostatin/activin also improves insulin sensitivity, reduces systemic inflammation and improves skeletal muscle atrophy in disease models.

A recent study demonstrated that intestinal dysbiosis and gastrointestinal barrier disruption may significantly contribute to sustaining low‐grade systemic inflammation in CKD.^19^ The human gut is heavily populated with myriad microorganisms.^20^ The normal gut microbial community forms a natural defence barrier and favourably influences the hosts' physiology, immune function, nutrition, inflammatory signalling and metabolism of lipids, bile acids, indole, choline, and short‐chain fatty acids.^21^ Various clinical and animal studies have demonstrated the critical role of gut microbiota in both health maintenance and disease pathogenesis.^22^ However, by altering the biochemical and biophysical milieu of the intestinal tract,^23^ advanced CKD results in intestinal epithelial barrier disruption and microbial dysbiosis.^24^ Recent studies have shown the effect of CKD on the gut microbiota, indicating an effect on the kidney–gut axis.^25^ CKD results in dysbiosis of the gut microbiota, leading to increased production of various uremic toxins, including LPS, p‐cresyl sulfate (p‐CS),^26^ trimethylamine and trimethylamine N‐oxide (TMAO), which accelerate the progression of CKD to end‐stage renal disease (ESRD) by intensifying inflammation^27^ and increasing the expression of inflammatory factors, such as IL‐1 and IL‐6. In CKD patients, the retention of uremic molecules and pro‐inflammatory cytokines is a key mechanism leading to oxidative stress and inflammation. In particular, two uremic toxins, indoxyl sulfate (IS) and LPS, are produced via fermentation by proteolytic bacteria in the gut and accumulate in the gut due to decreased glomerular filtration and reduced proximal tubular secretion.^28^ IS has been shown to be associated with aortic calcification and vascular smooth muscle cell proliferation in both rats and humans.^29^ Studies have shown that LPS can upregulate the expression of atrogin‐1 and MuRF1 by increasing the activity of NF‐κB and its downstream inflammatory mediators, such as TNF‐α, IL‐1β or the NLR family pyrin domains. This increase in activity lead to skeletal muscle atrophy, and a recent study showed that NLRP3 knockout protects mice from sepsis‐induced muscle wasting. These studies suggest an important role of NF‐κB‐related inflammatory mediators in ubiquitin–proteasome system‐associated muscle proteolysis. LPS reportedly induce muscle atrophy by increasing autophagosomes, atrogin‐1 and MuRF1 in C2C12 myotubes and in mouse skeletal muscle.^30^

The predominant gut bacteria in healthy individuals are affiliated with the phyla Bacteroidetes and Firmicutes (together accounting for 70–90% of gut bacteria), and Actinobacteria and Proteobacteria are also present. These four phyla constitute the core microbiome of the human gut.^31^, ^32^ *Bacteroides plebeius* is a human gut bacterium isolated from seaweed‐eating humans^31^ that belongs to the genus Bacteroidetes. A recent metagenomics analysis of gut microbiomes from healthy Japanese subjects identified a polysaccharide utilization locus (PUL) in the human gut bacterium *Bacteroides plebeius*; this locus contains CAZymes that appear to have been obtained via horizontal gene transfer from a marine bacterium. The PUL of *Bacteroides plebeius* may target the carbohydrates of red seaweeds such as porphyran and agarose.^33^, ^34^ Recent studies have shown that patients with CKD have a lower abundance of *Bacteroides plebeius* in the intestines compared with healthy individuals.^27^ The patients in the study ate normally according to the diet of patients with CKD. Studies have also shown that *Bacteroides plebeius* is an anti‐inflammatory bacterium.^32^ The current clinical trials have mostly focused on the microbiota differences between CKD patients and healthy individuals. No large‐scale clinical trial has explored the microbiota differences between well‐nourished and malnourished patients with CKD. However, the role and mechanism of action of *Bacteroides plebeius* in malnourished patients with chronic renal failure have not been studied.

In this study, we performed 16 S RNA sequencing of healthy people and malnourished patients with CKD (nutritional assessment of patients according to the Chinese Clinical Practice Guidelines for Nutritional Therapy of Chronic Kidney Disease (2021 Edition)) to elucidate the differences in the gut microbiota and Bacteroidetes between the two groups. We then established 5/6 Nx rats and treated these rats with *Bacteroides plebeius* to study the effect of this bacterium on CKD‐associated malnutrition. These studies allowed us to explore the mechanism through which *Bacteroides plebeius* reduces protein consumption in malnourished rats with CKD via the gut and kidneys and perhaps through the Mystn/ActRIIB/SMAD2 pathway.

## 2 MATERIALS AND METHODS

### 2.1 Participants

The participants were recruited at Southern Medical University Hospital of Integrated Traditional Chinese and Western Medicine from July 2019 to December 2019. Faecal samples from stage 3–4 CKD patients with CKD‐associated protein consumption (*n* = 47; the patients with malnutrition met at least three of the following diagnostic criteria: (1) albumin <38 g/L, (2) prealbumin <300 mg/L, (3) total cholesterol <2.59 mmol/L, (4) loss of muscle mass >5% over 3 months or > 10% over half a year, (4) decreased upper arm muscle circumference > 10% in the reference population, (5) BMI < 22 kg/m^2^ (under 65 years of age) or < 23 kg/m^2^ (over 65 years of age), (6) unexpected weight loss >5% over 3 months or > 10% over 6 months, (7) body fat percentage < 10%, (8) insufficient protein intake (DPI <0.8 g·kg^−1^·d^−1^) over at least 2 months, (9) insufficient energy intake (DEI < 25 kJ·kg^−1^·d^−1^) over at least 2 months) and healthy individuals (*n* = 49) were collected in the physical examination centre, and 16 S RNA sequencing was performed after the participants signed a written informed consent form. The study was approved by the Research Ethics Committee of the Hospital of Integrated Traditional Chinese and Western Medicine of Southern Medical University (reference: UHL 137056). To qualify for the CKD trial, the participants had to ① be 16–65 years of age, ② meet the 2012 CKD diagnostic criteria for the NKF of the United States and the diagnostic criteria for stages 3–4 CKD based on the Cockcroft‐Gault formula evaluation; ③ meet the malnutrition diagnostic criteria (refer to the International Society for Nephrology and Metabolism (ISRNM) CKD‐malnutrition diagnostic criteria) and the 2020 China Clinical Practice Guidelines for Nutritional Treatment of Chronic Kidney Disease; ④ have no serious systemic infection and have taken no antibiotics within the previous 3 months and ⑤ have the ability to give informed consent.

### 2.2 Animals and experimental design

All animal procedures were approved by the Institutional Animal Care and Use Committee (IACUC) of Southern Medical University with the certification number SCXK (Yue) 2016‐0041 and usage licence number L2018247. Male Sprague–Dawley rats at the age of 8 weeks (200–250 g) were purchased from the Experimental Animal Center of Southern Medical University, Guangzhou, China and were maintained in cages at 22–23°C under a 12‐h light/12‐h dark cycle with free access to water. The general overall appearance and emotional states of the rats were monitored. The rats were randomly divided into the sham operation (*n* = 4) and 5/6 nephrectomy (5/6 Nx) groups (*n* = 10). The sham‐operated rats were subjected to a sham operation, and one of the sham‐operated rats died unexpectedly. The rats in the 5/6 Nx group underwent surgical resection of the upper and lower thirds of the left kidney and right nephrectomy after 1 week. To decrease the bacterial load in the recipient rats, the rats in the 5/6 Nx group were treated with neomycin (all at 0.25 mg/day) and vancomycin (0.125 mg/day) once daily for 7 consecutive days by oral gavage.^35^ The 5/6 Nx rats were randomly divided into two groups: the 5/6 Nx model group (*n* = 5), which was treated with saline water irrigation of the stomach, and the *Bacteroides plebeius*‐treated group (*n* = 5). The rats were treated with *Bacteroides plebeius* by oral gavage at a dose of 2*10^8^ cfu/0.2 ml suspended in sterile anaerobic PBS (CT‐ *Bacteroides plebeius*). The rats in the control group were orally administered an equivalent volume of sterile anaerobic PBS containing a similar final concentration of glycerol.^36^ The general overall appearance of the rats was evaluated over time.

### 2.3 Bacteria

For the preparation of blood agar, 33.0 g of a mixture of 10.0 g of peptone, 3.0 g of beef extract, 5.0 g of sodium chloride and 15.0 g of agar with a pH of 7.3 ± 0.1 at 25°C was prepared in 1 L of distilled or deionized water. The solution was boiled until completely dissolved, divided into packs, sterilized by autoclaving at 121°C for 15 min, shaken well to prevent the agar from precipitating and solidifying on the bottom of the vessel and cooled to 50°C. After the addition of 5–10% defibrillated sheep blood, the mixture was well mixed and poured into the plate. *Bacteroides plebeius* (GDMCC 17135, Guangzhou, China) was cultured in a basal medium containing 0.25% w/v mucin with a pH of 6.5 at 37°C under strictly anaerobic conditions with a completely anaerobic gas bag (Japan MGC Anaero Pack®).

### 2.4 Blood and urine examination

At the end of the study, the rats were anaesthetised by intraperitoneal injection of sodium pentobarbital at a concentration of 40 mg/kg and fixed on the rat operating table. Blood was drawn, and 10 ml of blood was collected in a vacuum tube. The aortic blood of anaesthetised rats was used for measurements of the serum creatinine (SCr) (C011‐2‐1JianChen Nan), blood urea nitrogen (BUN) (C013‐1‐1JianChen Nanjing), serum albumin (A028‐2‐1JianChen Nanjing), serum prealbumin (HQ‐30565), transferrin (HQ‐30578), IL‐1 (JM‐10482R1), IL‐6 (HQ‐30646) and LPS (HQ‐30079) levels.

The 24‐h urine samples were collected using metabolic cages, and urinary protein excretion was tested using a commercial kit (C035‐2‐1, JianChen Nanjing) according to the instructions of the manufacturer.

### 2.5 16 S rRNA sequencing

#### 2.5.1 DNA extraction and PCR amplification

Faecal samples were collected using standardized collection procedures and were selected at random. Microbial community genomic DNA was extracted from the samples using the E.Z.N.A.® soil DNA Kit (Omega Bio‐Tek, Norcross, GA, USA) according to the manufacturer's instructions. The DNA extract was evaluated on a 1% agarose gel, and the DNA concentration and purity were determined with a NanoDrop 2000 UV–vis spectrophotometer (Thermo Scientific, Wilmington, USA). An OD260/280 ratio in the range of 1.8–2.0 indicates sufficient quality. A ratio lower than 1.8 indicates impurities, such as protein and phenol, in the sample. The hypervariable V3–V4 region of the bacterial 16 S rRNA gene was amplified with the primer pair 338F (5'‐ACTCCTACGGGAGGCAGCAG‐3') and 806R (5'‐GGACTACHVGGGTWTCTAAT‐3') using an ABI GeneAmp® 9700 PCR thermocycler (ABI, CA, USA). PCR amplification of the 16 S rRNA gene was performed as follows: initial denaturation at 95°C for 3 min, 27 cycles of denaturing at 95°C for 30 s, annealing at 55°C for 30 s and extension at 72°C for 45 s, a single extension at 72°C for 10 min and ending at 4°C. The PCR mixtures contained 4 μl of 5× TransStart FastPfu buffer, 2 μl of 2.5 mM dNTPs, 0.8 μl of forward primer (5 μM), 0.8 μl of reverse primer (5 μM), 0.4 μl of TransStart FastPfu DNA Polymerase, 10 ng of template DNA, and ddH_2_O up to 20 μl. The PCR product was extracted from a 2% agarose gel, purified using the AxyPrep DNA Gel Extraction Kit (Axygen Biosciences, Union City, CA, USA) according to the manufacturer's instructions and quantified using a Quantus™ Fluorometer (Promega, USA). For Illumina MiSeq sequencing, the purified amplicons were pooled in equimolar amounts and paired‐end sequenced on an Illumina MiSeq PE300 platform/NovaSeq PE250 platform (Illumina, San Diego, CA, USA) according to the standard protocols of Majorbio Bio‐Pharm Technology Co., Ltd. (Shanghai, China). The raw reads were deposited into the NCBI Sequence Read Archive (SRA) database.

### 2.6 Processing of sequencing data

The raw 16 S rRNA gene‐sequencing reads were demultiplexed, quality‐filtered using fastp version 0.20.0^37^ and merged using FLASH version 1.2.7^38^ with the following criteria. (i) The 300‐bp reads were truncated at any site receiving an average quality score lower than 20 over a 50‐bp sliding window, and the truncated reads shorter than 50 bp were discarded. Reads containing ambiguous characters were also discarded. (ii) Only overlapping sequences longer than 10 bp were assembled according to their overlapping sequence. The maximum mismatch ratio of the overlap region was 0.2. Reads that could not be assembled were discarded. (iii) Samples were distinguished according to the barcode and primers, and the sequence direction was adjusted based on exact barcode matching and two nucleotide mismatches in primer matching.

Operational taxonomic units (OTUs) with a 97% similarity cut‐off of^26^ were clustered using UPARSE version 7.1,^39^ and chimeric sequences were identified and removed. The taxonomy of each OTU representative sequence was analysed using RDP Classifier version 2.2^40^ against the 16 S rRNA database (e.g. Silva v138) based on a confidence threshold of 0.7.

### 2.7 Histological analysis

The rats were anaesthetised, and the intestinal tissue and gastrocnemius muscle were carefully dissected and immersed in 4% paraformaldehyde overnight at 4°C. After embedding in paraffin, the tissue was cut into thin slices (4 μm). Haematoxylin–eosin (HE) staining of the muscle and intestinal tissues was performed. For immunofluorescence (IF) analysis, the colon sections were blocked with 5% BSA for 1 h and incubated with antibodies against ZO‐1 and Calludin‐2. The primary antibody (1:200; Affinity) was incubated overnight at 4°C, and the Cy3‐labelled secondary antibody was incubated at room temperature for 1 h. The nucleus was stained with DAPI (Servicebio). Images were captured with an MVX20 microscope (Olympus). For imaging at different time points, a confocal microscope (Zeiss, LSM800), ZEN software and Microsoft PowerPoint were used for image processing.

### 2.8 Polymerase chain reaction

Total RNA was extracted from skeletal muscle tissue using TRIzol reagent (Vazyme Biotech Co., Ltd.) and converted into cDNA using a HiScript® III RT SuperMix for qPCR (+gDNA wiper) kit (Vazyme Biotech Co., Ltd.). qPCR was performed using ChamQ Universal SYBR qPCR Master Mix (Vazyme Biotech Co., Ltd.). All primers used are shown in Table S1. The relative mRNA level was calculated by normalization to the GAPDH level (B661304, Sangon Biotech, Shanghai, China). The analyses of relative gene expression were based on the fold change (2‐ΔΔCt method).

### 2.9 Western blotting

According to the manufacturer's instructions, the protein was extracted using a whole‐cell lysis assay (KeyGEN, Nanjing, China). Proteins were separated using SDS–PAGE and transferred to PVDF membranes (Millipore, Bedford, MA, USA). The membranes were blocked in 5% BSA for 1.5 h and then incubated with primary antibodies targeting the following proteins: claudin‐2 (AF0127; 1:1000; Affinity), ZO‐1 (AF5145; 1:1000; Affinity), MURF‐1 (AF21074; 1:1000; Affinity), MAFBX (AF67172; 1:1000; Affinity), SMAD2 (AF0131; 1:1000; Affinity), MSTN (ab4567 1:1000; Abcam), ActRIIB (ab272869; 1:1000; Abcam) and GAPDH‐HRP (D16H11, 1:1000; CST). After washing with TBS‐T, the membrane was incubated with a secondary antibody (SA00001‐2, 1:8000; Proteintech) at 4°C for 2 h unless the GAPDH‐HRP antibody was used. The ECL system (Affinity, Jiangsu, China) was used for the detection of protein signals.

### 2.10 Statistical analyses

The statistical analyses were performed with SPSS (IBM SPSS Statistics for Windows, version 20; IBM Corp., Armonk, NY, USA). Comparisons between groups with one and two independent variables were performed by one‐way anova and two‐way anova, respectively, followed by the least significant difference (LSD) post hoc test. The following significance levels were used for comparisons between independent groups: *p < 0.05, **p < 0.01 and ***p < 0.001 versus the control group and ^#^ *p* < 0.05, ^##^ *p* < 0.01 and ^###^ *p* < 0.001 versus the 5/6 Nx model.

## 3 RESULTS

### 3.1 Gut Microbiota of CKD patients with muscle wasting

**FIGURE 1:**
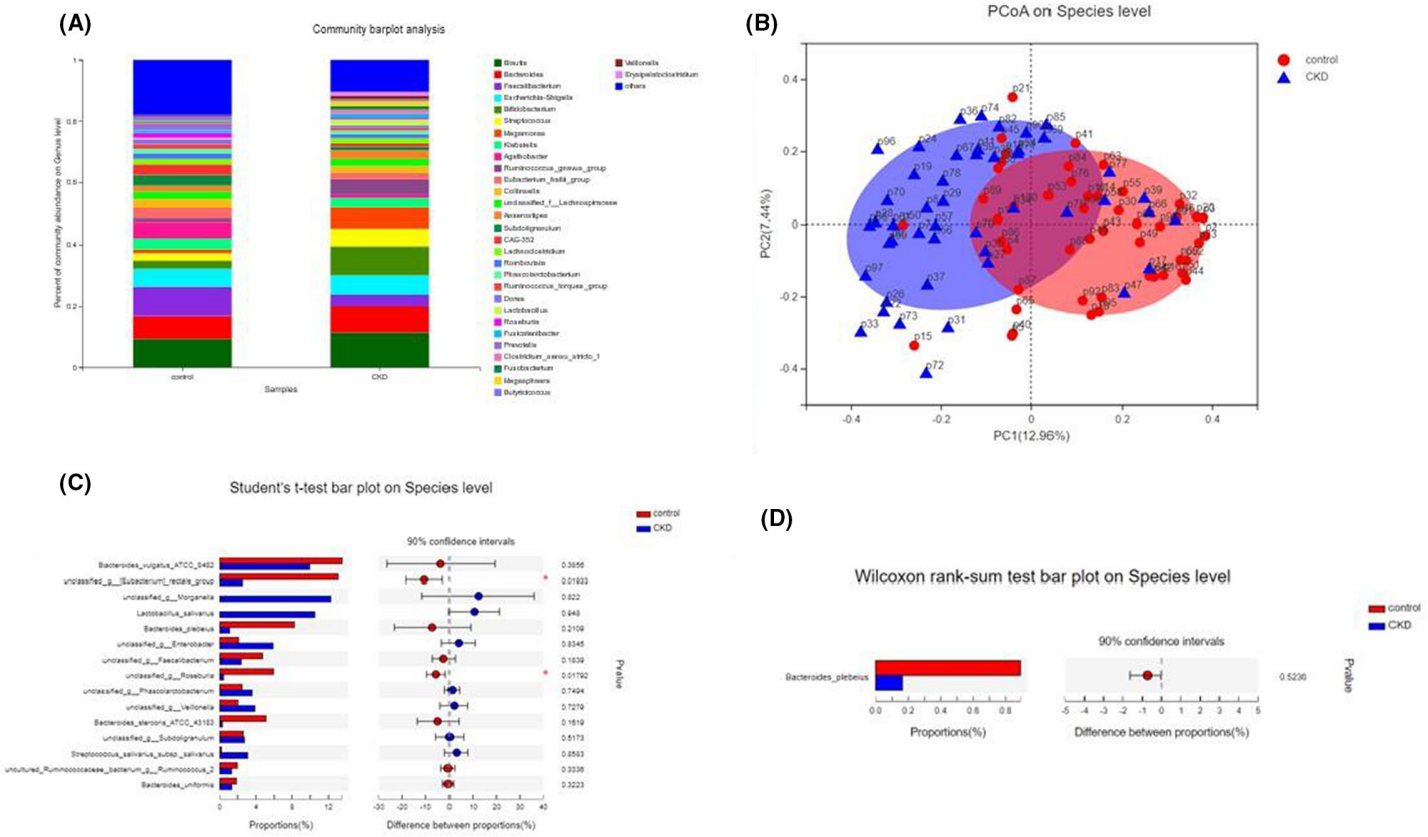
Difference in intestinal microecology between malnourished patients with CKD and healthy individuals. (A) Heatmap of the microbial community abundance distribution at the phylum level. The top 30 species in terms of total abundance are presented. (B) PCoA plot of the Bray–Curtis distance at the operational taxonomic unit (OTU) level. (C) Student's t bar plot at the species level. (D) The difference in the *Bacteroides plebeius* abundance between the two groups.

The sequence of the 16 S rRNA gene V3/V4 variable region in stool samples was analysed. Faecal metagenomic sequencing data can be obtained from the National Center for Biotechnology Information (NCBI) database with an access code. To illustrate the richness and diversity of the community, we used the Chao, Shannon, Simpson, Sobs and Npshannon diversity indices. The diversity and abundance of the gut microbes in CKD patients with malnutrition were lower than those found in healthy individuals (Figure 1A). A principal coordinate analysis (PCoA) revealed that CKD‐malnourished and healthy individuals presented better intragroup clustering (Figure 1B). Second, we assessed the differences in the composition of the flora between the two groups and found that *Bacteroides plebeius* was significantly more abundant in the microbial community of healthy people than in that of CKD‐malnourished patients who followed the dietary guidelines regarding the daily CKD diet (Figure 1C,D).

### 3.2 *Bacteroides plebeius* improved renal function

**FIGURE 2:**
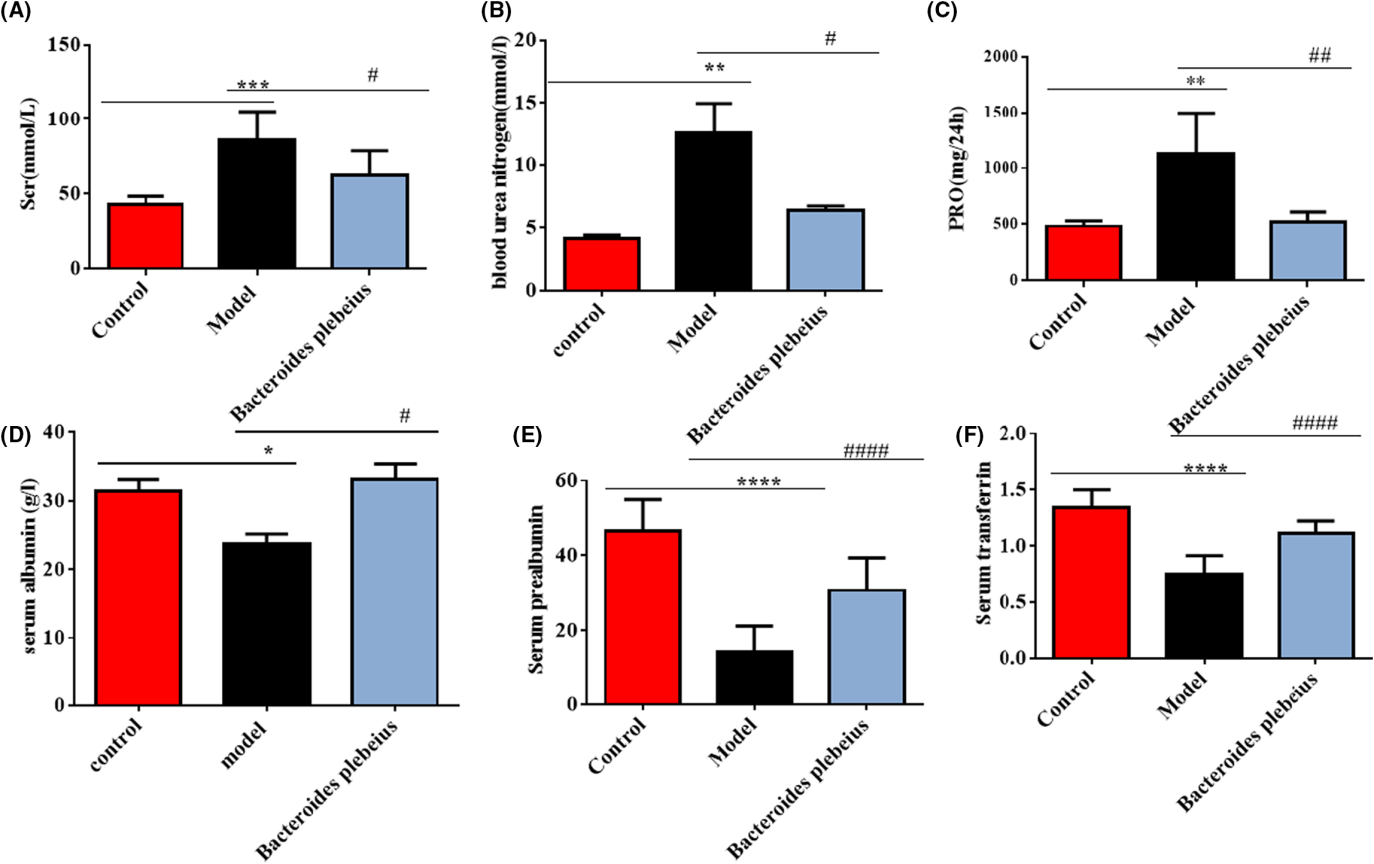
*Bacteroides plebeius* reduces renal function parameters and body weight after 5/6 Nx. (A) *Bacteroides plebeius* reduced the serum creatinine (Scr) level of CKD rats. (B) *Bacteroides plebeius* reduced the serum blood urea nitrogen (BUN) level of CKD rats (*n* = 5). (C) *Bacteroides plebeius* reduced the 24‐h urine protein level of CKD rats. (D) *Bacteroides plebeius* improved the serum albumin (ALB) level of CKD rats. (E) *Bacteroides plebeius* increased the transferrin (TF) levels of CKD rats. (F) *Bacteroides plebeius* improved the serum prealbumin protein (PAB) levels of CKD rats. All the data are expressed as the means ± SDs. ^#^ *p* < 0.05 and ^##^ *p* < 0.01 vs. the 5/6 Nx group, and *p < 0.05 and **p < 0.01 vs. the sham group.

The renal function results found for the sham operation, 5/6 Nx model, and *Bacteroides plebeius* treatment groups are shown in Figure 2. The BUN, SCr, and 24‐h urine protein levels in the sham operation group were within the normal range, whereas those in the CKD group were elevated. However, the administration of *Bacteroides plebeius* for 8 weeks significantly reduced the BUN, SCr and 24‐h urine protein levels (Figure 2A–C). In addition, *Bacteroides plebeius* increased the serum ALB transferrin (TF) and serum prealbumin (PAB) levels (Figure 2D–F).

### 3.3 *Bacteroides plebeius* inhibited CKD‐induced muscle atrophy

**FIGURE 3:**
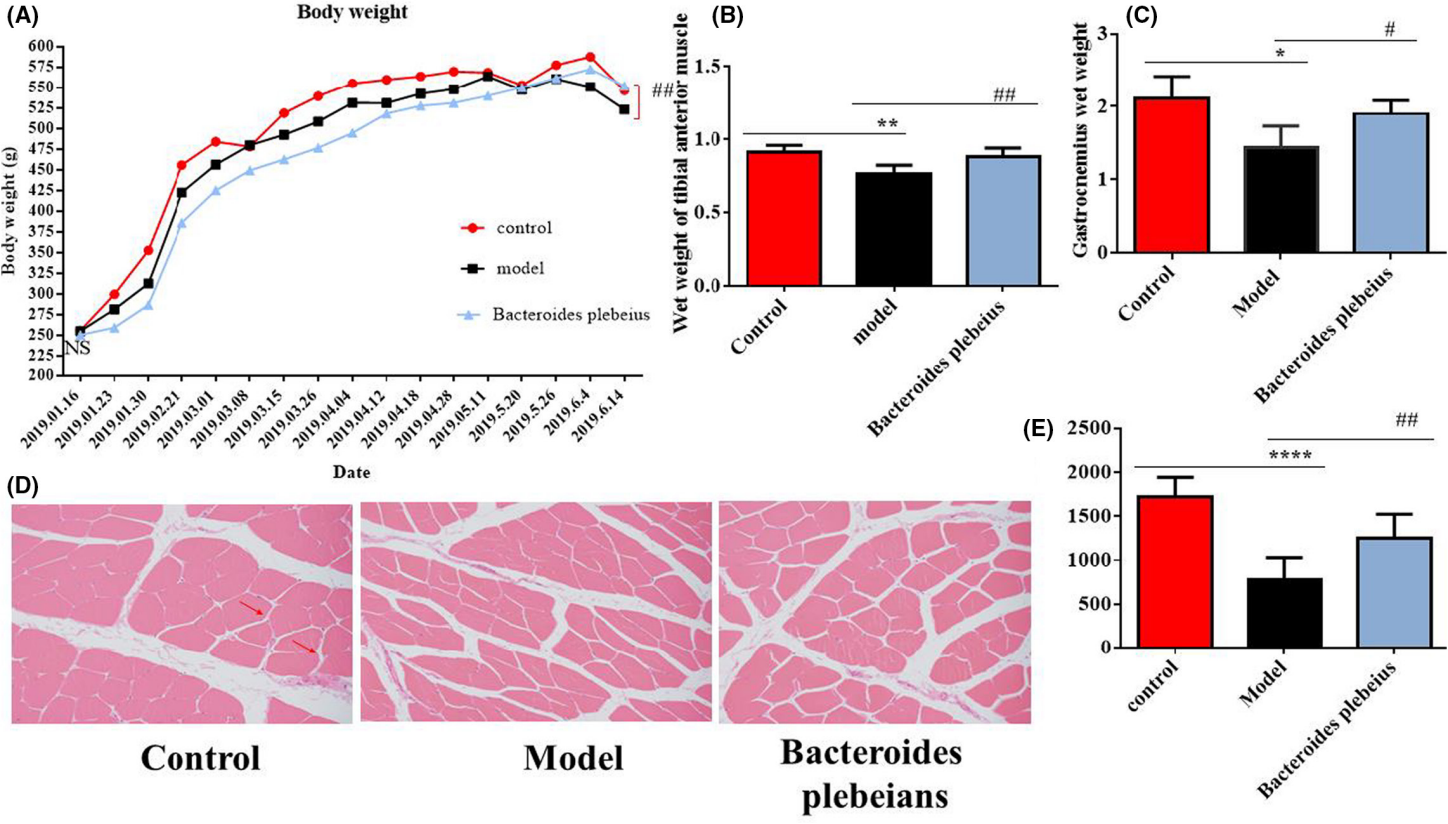
*Bacteroides plebeius* significantly improves muscle atrophy in CKD‐malnourished rats. (A) *Bacteroides plebeius* the increased body weight of CKD‐malnourished rats. (B) *Bacteroides plebeius* increased the wet weight of the tibial anterior muscle of CKD‐malnourished rats (*n* = 5). (C) *Bacteroides plebeius* increased the gastrocnemius wet weight of CKD‐malnourished rats. (D, E) Paraffin sections from tibial anterior muscle tissues were stained with H&E and observed under a microscope (200× magnification). The red arrows indicate myofibres affected by atrophy. The cross‐sectional area of the muscle fibres of different groups was measured using ImageJ. All the data are expressed as the means ± SDs. ^#^ *p* < 0.05 and ^##^ *p* < 0.01 vs. the 5/6 Nx group and *p < 0.05 and **p < 0.01 vs. the sham group.

The body weight and muscle fibre area were measured as direct indicators of muscle atrophy. Compared with that of rats in the sham operation group, the weight of the rats in the 5/6 Nx model was significantly reduced, and that of the rats increased after *Bacteroides plebeius* treatment (Figure 3A). Compared with that of the sham operation group, the wet weights of the gastrocnemius (Gastroc) and tibial anterior muscle (TA) of the 5/6 Nx model were significantly lower, whereas those of the *Bacteroides plebeius* treatment group were increased (Figure 3B,C). The gastrocnemius and tibial anterior muscles of the *Bacteroides plebeius* treatment group were larger than those of the 5/6 Nx group but smaller than those of the control group. The cross‐sectional area of muscle fibres is often used to describe muscle atrophy, which allows for avoiding potential confounding factors associated with changes in the extracellular space. We observed that the average cross‐sectional area of 5/6 Nx model rats was smaller than that of sham rats. Similarly, the cross‐sectional area after treatment with *Bacteroides plebeius* was larger than that of the 5/6 Nx model but smaller than that of the sham operation group (Figure 3D,E).

### 3.4 Effect of *Bacteroides plebeius* on the Gut Microbiota of 5/6 Nx Rats

**FIGURE 4:**
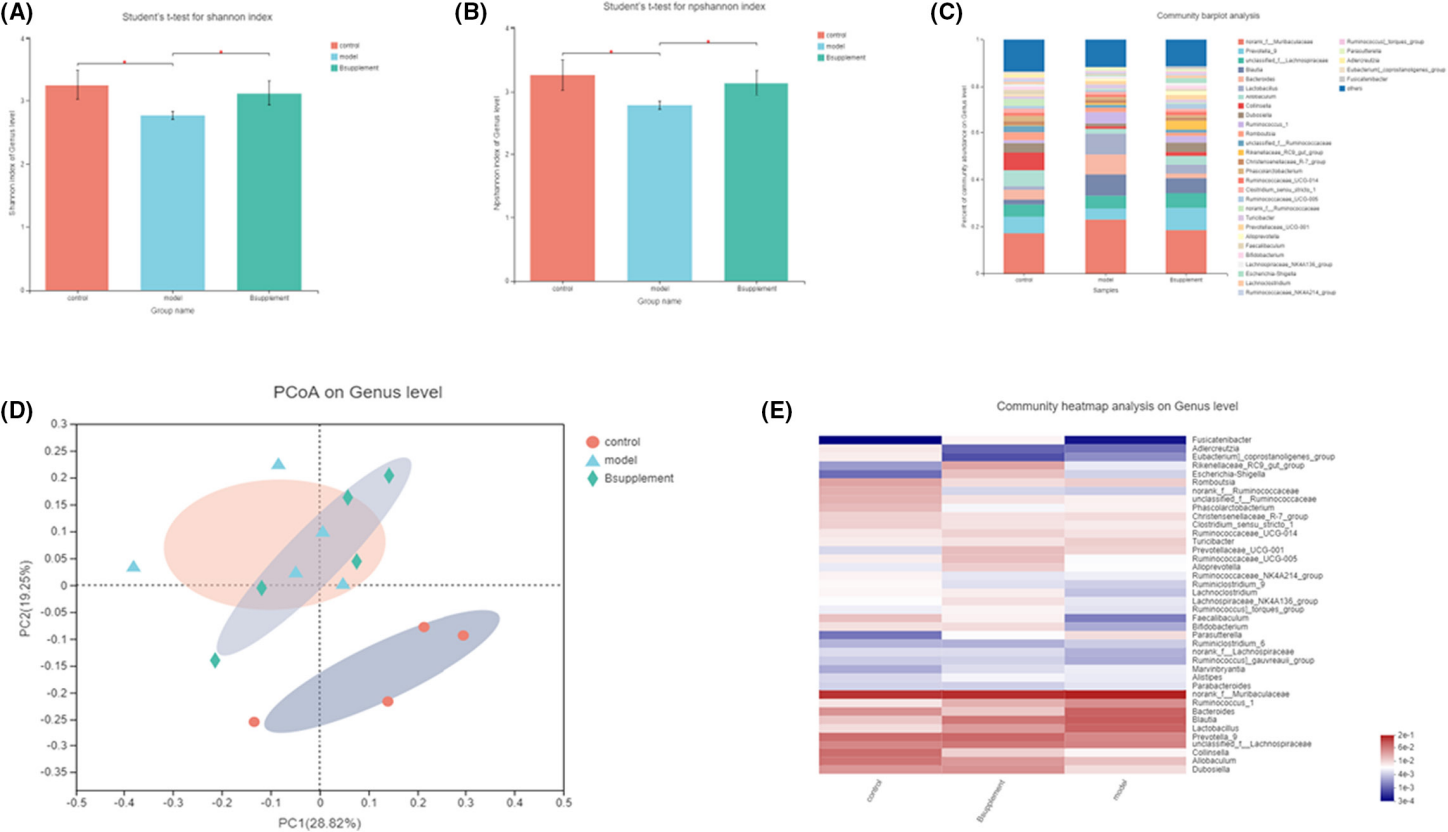
Effect of *Bacteroides plebeius* on the intestinal microecology of CKD‐malnourished rats. (A) Sobs index values of each group. (B) Npshannon index values of each group. (C) PCoA plot of the Bray–Curtis distance at the operational taxonomic unit (OTU) level. (D) Barplot analysis of the relative abundance of the community at the genus level. Genera with an abundance <0.01 (1%) were included in the “other” category. A heatmap of the microbial community abundance distribution at the phylum level is presented. The top 50 species in terms of total abundance are shown.

The sequence of the V3/V4 variable region of the 16 S rRNA gene in faecal samples was analysed. Faecal metagenomic sequencing data are available from the National Center for Biotechnology Information (NCBI) database with accession codes. To illustrate the richness and diversity of the community, we used the Chao, Shannon, Simpson, Sobs and Npshannon diversity indices. Compared with those of the flora of the sham operation group, the abundance and diversity of the flora of the 5/6 Nx group were reduced and improved after *Bacteroides plebeius* intervention (Figure 4A,B). A PCoA revealed that the sham operation group, 5/6 Nx group and *Bacteroides plebeius* intervention group exhibited better clustering. In contrast to that of the 5/6 Nx model, the clustering of the *Bacteroides plebeius* group began to approach that of the blank group (Figure 4C). We conducted barplot, Circos (Figure S1), and heatmap (Figure 4D) analyses. A Circos diagram was used to visualize the associations between the bacterial communities of different samples in terms of abundance at the genus level, and the results were consistent with the barplot analysis.

### 3.5 Effects of *Bacteroides plebeius* on the intestinal barrier

**FIGURE 5:**
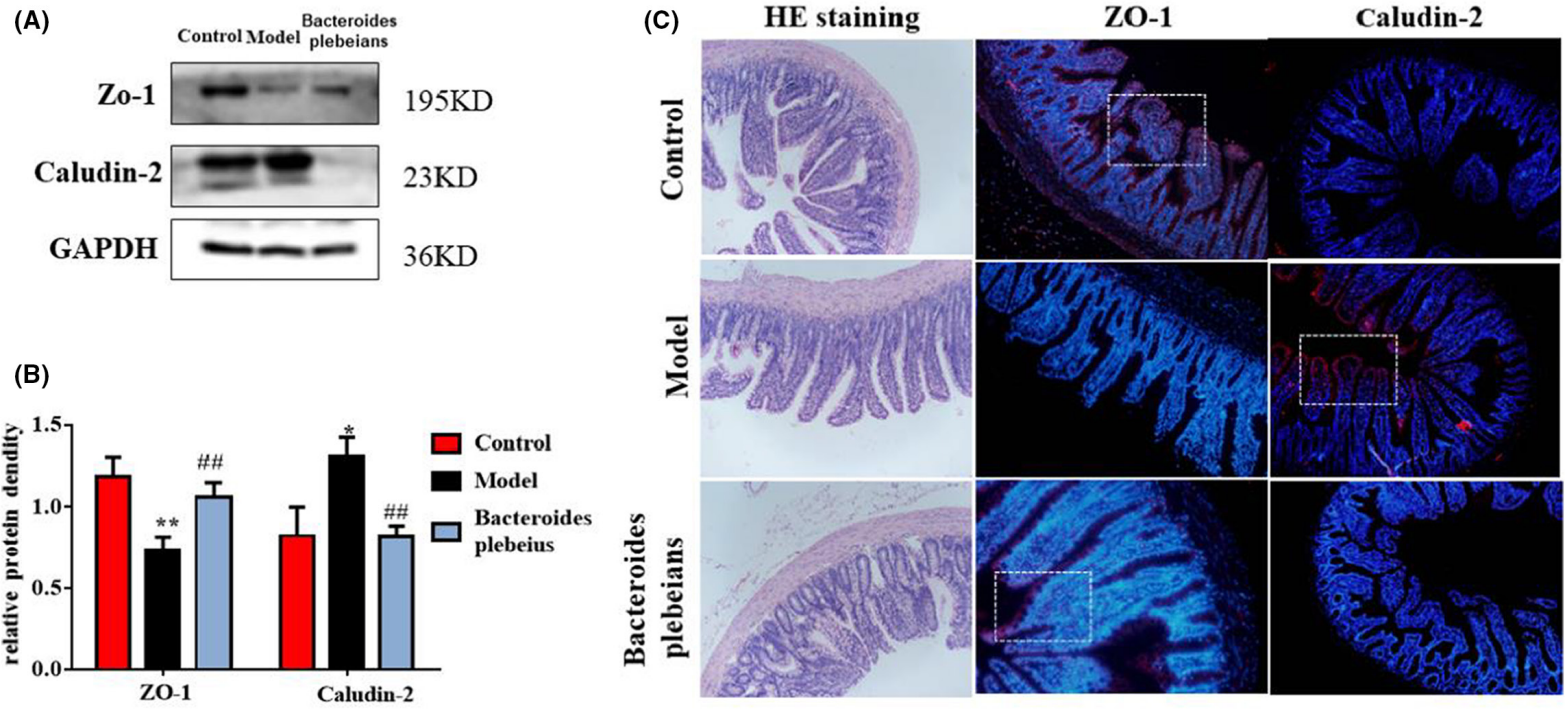
Effect of *Bacteroides plebeius* on the intestinal barrier in CKD‐malnourished rats. (A) Western blot analysis of ZO‐1 and claudin‐2 in the intestine. (B) Protein quantification by Western blotting. (C) HE staining of rat intestinal tissues (scale bar = 100 μm) and ZO‐1 and claudin‐2 expression in the intestine, as determined by immunofluorescence staining (200×) using anti‐ZO‐1 and anti‐claudin‐2 (green) antibodies. The nuclei were detected via DAPI staining (blue). All the data are shown as the means ± SEMs (*n* = 5 for each group) *p < 0.05 and **p < 0.01 versus the control group, and ^#^ *p* < 0.05 and ^##^ *p* < 0.01 versus the 5/6 Nx model.

Histological and Western blot analyses showed that the integrity and tight connectivity of the intestine of 5/6 Nx rats were destroyed. HE staining of the intestines showed an irregular cell arrangement in the 5/6 Nx group, and oedema was observed in the villi (villus width). Compared with the intestinal mucosa of the 5/6 Nx model, the intestinal mucosa of the sham operation group and the *Bacteroides plebeius* group exhibited a more complete structure and less inflammatory infiltration of the crypts, fewer villi (Figure 5A), and higher ZO‐1 protein levels were detected in the *Bacteroides plebeius* group (Figure 5B,C). In addition, the expression of the claudin‐2 protein was lower in the *Bacteroides plebeius* group. Immunofluorescence staining also revealed the same protein expression pattern.

### 3.6 Influence of *Bacteroides plebeius* on inflammatory factors and endotoxins and correlations between inflammatory factors and the intestinal microbiota

**FIGURE 6:**
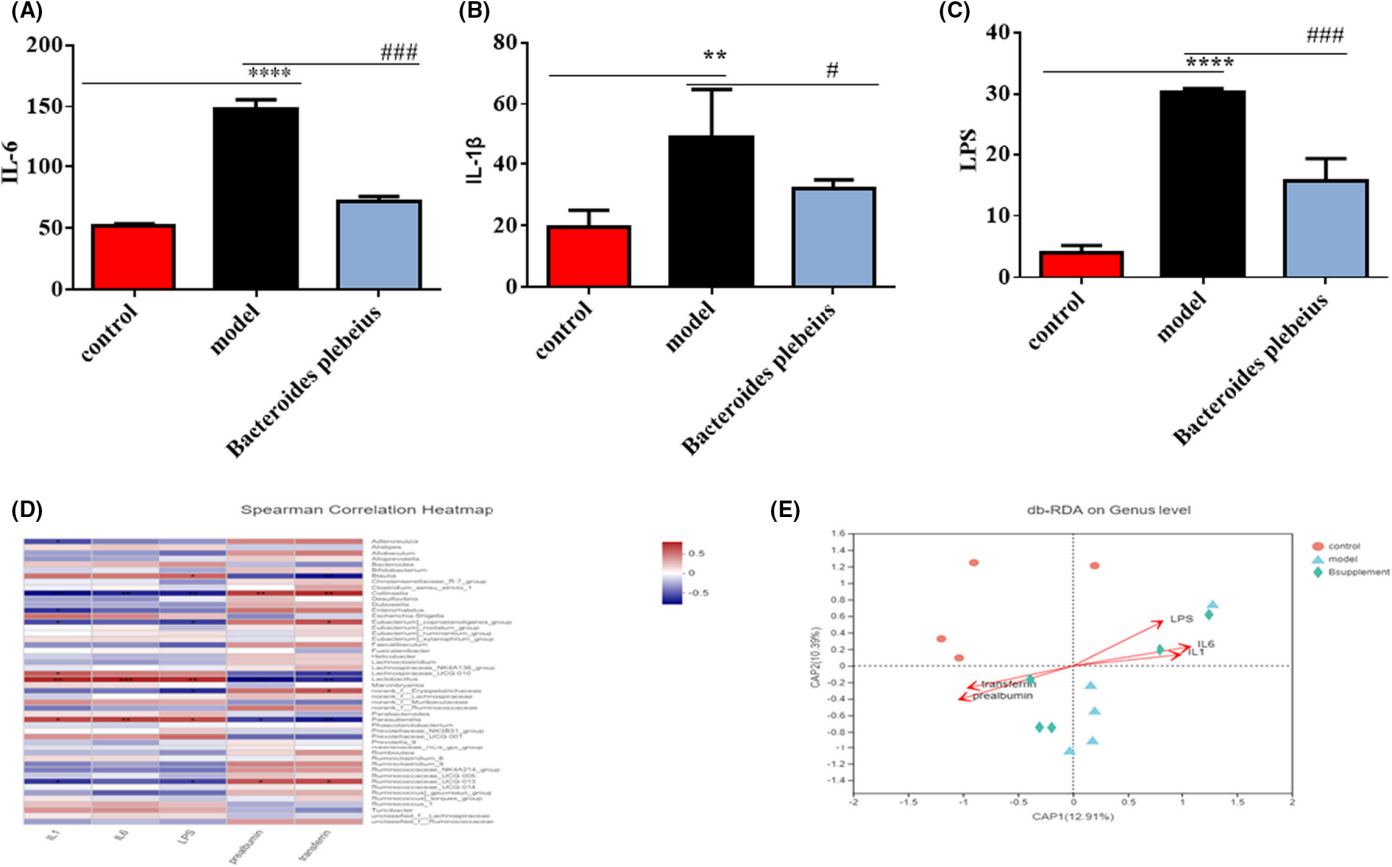
Effect of *Bacteroides plebeius* on inflammation and endotoxin levels in 5/6 Nx rats and correlation analysis among inflammatory factors, endotoxins, and the intestinal flora. (A) IL‐1β, (B) IL‐6, and (C) LPS. (D) Correlation analysis of IL‐1β, IL‐6, and LPS with the intestinal flora. (E) Correlation analysis of IL‐1β, IL‐6, and LPS in each group. The bars represent the means ± SEs of each group (*n* = 5). *p < 0.05, **p < 0.01, and ***p < 0.001 versus the control group, and ^#^ *p* < 0.05, ^##^ *p* < 0.01, and ^###^ *p* < 0.001 versus the 5/6 Nx model.

To investigate the inflammatory alterations in rats, we detected the levels of the inflammatory factors IL‐1β, IL‐6 and LPS in each group. As expected, the levels of all factors were notably increased in the serum of the 5/6 Nx group, which indicated that inflammation plays an important role in the development of CKD. Consistently, *Bacteroides plebeius* markedly decreased the levels of IL‐1β, Il‐6 and LPS in the serum of the 5/6 Nx rats (Figure 6A–C), which indicated that *Bacteroides plebeius* could inhibit the inflammatory response in CKD.

The correlation analysis of the intestinal microecology and IL‐1β, IL‐6 and LPS at the genus level revealed that the Parasutterella, Lactobacillus and Lachnospiraceae abundances were positively correlated with the IL‐1, IL‐6 and LPS levels and that the Eubacterium and Ruminococcaceae abundances were negatively correlated with the IL‐1, IL‐6 and LPS levels (Figure 6D). In the db‐RDA, at the OTU level, 5/6 Nx rats exhibited a positive correlation with the IL‐1, IL‐6 and LPS levels. The sham operation and *Bacteroides plebeius* treatment were negatively correlated with IL‐1, IL‐6 and LPS levels (Figure 6E).

### 3.7 *Bacteroides plebeius* improves muscle consumption in CKD‐malnourished rats by improving insulin resistance

**FIGURE 7:**
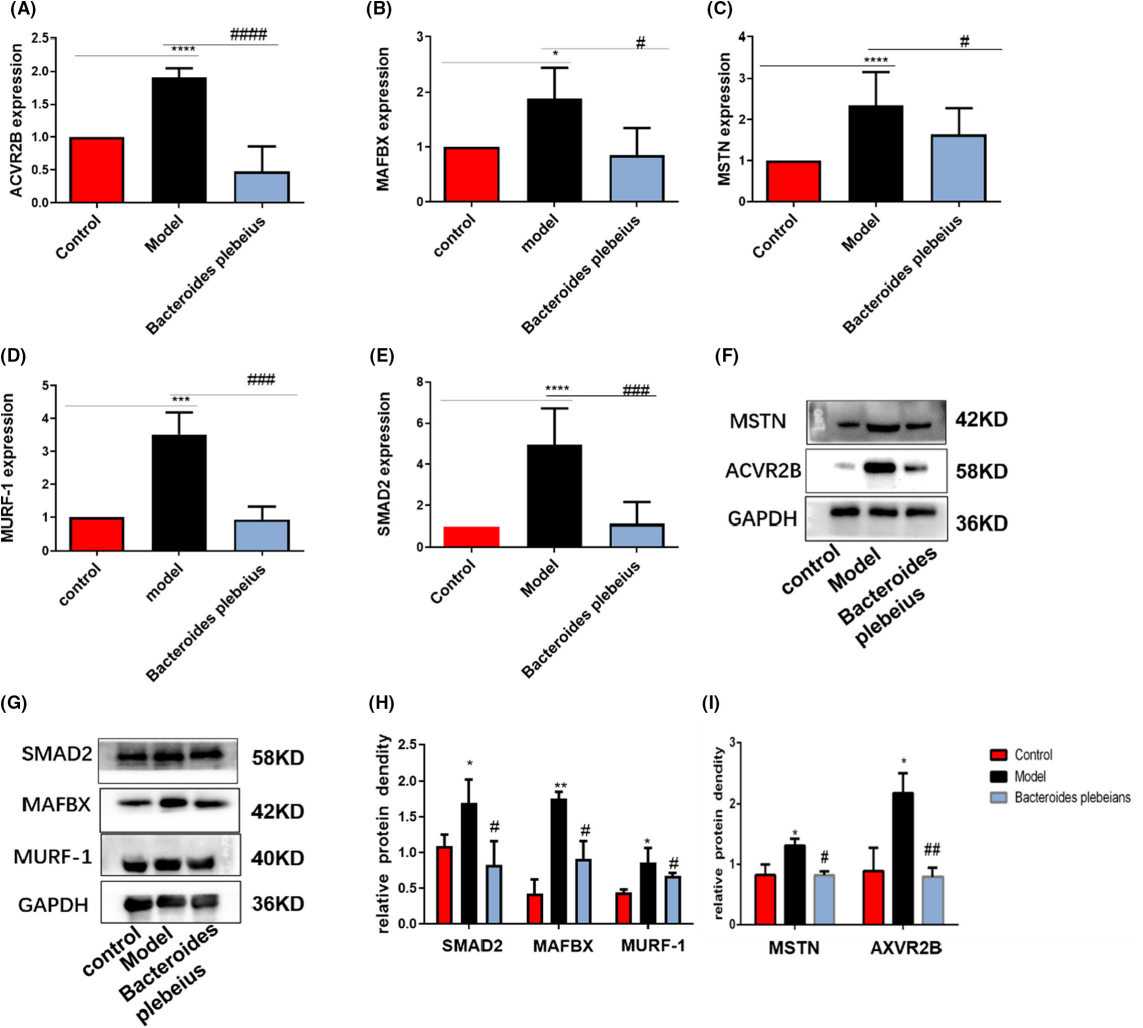
*Bacteroides plebeius* inhibits myostatin‐mediated insulin resistance‐related pathways in the muscles of CKD rats. (A–E) mRNA levels of MSTN, SMAD2, ACVR2B, MAFBx, and MuRF‐1 determined by qPCR. (F, G) Western blot analysis of MSTN, SMAD2, ACVR2B, MAFBx, and MuRF‐1 protein levels in the gastrocnemius muscle. (H, I) ImageJ analysis of the MSTN, SMAD2, ACVR2B, MAFBx, and MuRF‐1 protein expression levels in the gastrocnemius muscle. All the data are expressed as the means ± SEMs (*n* = 5 in each group). *p < 0.05 and **p < 0.01 versus the control group, and ^#^ *p* < 0.05 and ^##^ *p* < 0.01 versus the 5/6 Nx model.

Compared with the CKD rats that did not receive *Bacteroides plebeius*, the CKD rats that received *Bacteroides plebeius* for 8 weeks showed significantly increased muscle protein synthesis and significantly reduced protein degradation. We found that *Bacteroides plebeius* inhibited the expression of ubiquitin E3 ligases (including MAFBx and MuRF‐1). We examined whether the beneficial effects of *Bacteroides plebeius* on muscle atrophy are related to insulin resistance. Western blotting showed that treatment with *Bacteroides plebeius* reduced the expression of MSTN, SMAD2 and ACVR2B, and these expression levels were increased in rats with chronic renal failure‐associated protein depletion (Figure 7A,B). The qPCR results confirmed this trend (Figure 7C–G).

## 4 DISCUSSION

Malnutrition is characterized by abnormal reductions in the body's protein mass and energy reserves,^41^ and skeletal muscle atrophy, a hallmark of protein consumption, is prevalent in patients with CKD. These conditions are linked through a complex set of mechanisms, including inadequate nutritional intake, which is an important cause of morbidity and mortality.^42^ The gut microbiota has recently emerged as a key factor regulating host metabolism and energy balance.^43^ Disturbances in the normal gut microbiota, which are commonly referred to as gut dysbiosis, have been implicated in the pathogenesis of malnutrition in various disease states.^44^ CKD profoundly alters the composition and function of the gut microbiota.^45^ The human gut microbiota can influence the host physiology by modulating multiple processes, including nutrient absorption, inflammation, oxidative stress, immune function and anabolic homeostasis.^46^, ^47^ Consistent with other findings, our clinical findings suggest that gut microbiota homeostasis is disrupted and that the gut microbiota abundance and richness are reduced in CKD‐malnourished patients compared with healthy individuals. *Bacteroides plebeius* is abundant in healthy individuals. However, the sample size of our clinical patients was small, and we only verified the results reported by other research groups. We will expand the sample size to conduct a large‐sample clinical study in the future. The findings reported by Kiyotaka Uchiyama et al^48^ suggest that changes in the gut microbiota composition may promote chronic inflammation and anabolic resistance, which ultimately leads to decreased muscle mass, impaired muscle function and poor clinical outcomes.^49^ This finding was demonstrated by our animal experimental results, which revealed changes in the gut microbiota composition of 5/6 Nx rats. Intestinal microecological homeostasis was disrupted, and the rats exhibited signs of malnutrition, such as weight loss and decreased serum albumin and transferrin levels. This finding is consistent with the results from clinical studies.

CKD patients are in a state of microinflammation for a long time.^50^ Changes in the gut microbiome resulting in a significant reduction in gut permeability.^51^ Endotoxins enter the circulation and exacerbate the body's inflammatory state.^52^ Our results showed that CKD rats with skeletal muscle atrophy exhibit increased intestinal mucosal permeability and significantly decreased ZO‐1 protein expression levels. Studies have shown that claudin‐2 deficiency slows the progression of colitis and leaky barrier defects, and claudin‐2 is known to lead to the sufficient formation of cation‐selective channels to convert “tight” junctions into leaky channels.^53^ Our findings suggest that *Bacteroides plebeius* reduces claudin‐2 expression, increases intestinal mucosal tightness and partially restores the intestinal mucosal barrier. However, some experimental results suggest that claudin‐2 exerts a protective effect on intestinal mucosal tightness in some diseases, and this topic thus needs to be further researched. Disease‐related intestinal mucosal barrier dysfunction may be associated with the gut–kidney–muscle axis, which plays a central role in facilitating the entry of microorganisms. Gut microbial products or microorganisms enter the systemic circulation and help activate inflammatory responses and induce immune system diseases.^54^ Our pathological findings from 5/6 Nx model rats suggest that disease‐associated changes in the gut microbiota composition promote intestinal mucosal permeability. This phenomenon results in increased systemic absorption of bacterial products, including lipopolysaccharides, which activate an inflammatory response and ultimately lead to elevated circulating levels of pro‐inflammatory cytokines such as IL‐6 and interleukin‐1. The IL‐6 levels have been associated with weight loss in certain human cancers. A long‐term microinflammatory state occurred following the alteration in the gut microbiota homeostasis in 5/6 Nx rats. Our results show that enhanced intestinal permeability leads to increases in the level of the intestinal microbial product LPS in vivo and thus aggravates the inflammatory state of the body. Disease states are associated with changes in the gut microbiome and significantly elevated levels of IL‐1 and IL‐6. At the chronic inflammatory state, the level of the endotoxin syndrome‐associated toxin LPS was significantly increased in the 5/6 Nx model. Many pro‐inflammatory cytokines, such as TNF‐α, IL‐6 and IL‐1, reportedly promote muscle proteolysis,^55^ and causative factors have been shown to stimulate muscle proteolysis and inhibit protein synthesis, which leads to a decreased muscle mass.^54^ Inflammation has emerged as a key biological event leading to muscle wasting.^52^, ^56^, ^57^ Inflammation continuously promotes the expression of the ubiquitin E3 ligase MuRF‐1^58^, ^59^ and thereby increases muscle proteolysis through the ubiquitin–proteasome system. Inflammation can promote the expression of specific genes (such as MuRF‐1 and MAFBX).^60^ This finding is consistent with our results that the protein expression levels of MuRF‐1 and MAFBX are significantly increased in 5/6 Nx rats and that activation of myostatin/activin signalling is critical for triggering accelerated muscle catabolism, which in turn contributes to muscle loss in various disease states. The binding of myostatin and activin to the ActRIIB receptor complex on the muscle cell membrane results in the activation of Smad2/3‐mediated transcription, which in turn stimulates activation of the A‐SMAD signalling pathway associated with microinflammatory states. Both myostatin and activin A are upregulated in patients with various types of malignancies, and both the loss and increased expression of myostatin exert significant effects on muscle mass. ActRIIB decoy receptor therapy prevents the development of cachexia, increases muscle function and even prolongs survival in several cancer models. Furthermore, the initiation of this treatment after the development of cachexia completely reverses not only the loss of skeletal muscle but also the loss of cardiac mass. Our findings suggest that 5/6 Nx rats exhibit reductions in the skeletal muscle weight and volume and increased ActRIIB and A‐SMAD protein expression and that these effects improve after the intervention. Studies have shown that the levels of circulating microbiota‐derived indoxyl sulfate are positively correlated with the expression of myostatin and atrogin‐1, which are two major negative regulators of skeletal muscle mass. These studies highlight the role of gut microbiota‐derived metabolites in promoting skeletal muscle anabolism. The results show that 5/6 Nx rats exhibit increased serum levels of the circulating microbial derivative LPS and increased expression of two major negative regulators of skeletal muscle mass, MAFBX and MURF1 and improvements in these variables were observed after *Bacteroides plebeius* intervention. The findings suggest that *Bacteroides plebeius* restores CKD‐malnourished gut microbiota homeostasis, restores the intestinal barrier function and reduces the production of circulating microbiota‐derived metabolites involved in skeletal muscle synthesis and metabolism, which results in alleviation of the body's microinflammatory state and improvements in the body's inflammatory state and in skeletal muscle atrophy caused by the inflammatory state. Restoring the myostatin/activin B‐SMAD signalling pathway improves the nutritional status of CKD protein‐deficient rats. However, we only explored the possible mechanism through which *Bacteroides plebeius* improves skeletal muscle atrophy in CKD and did not verify this possibility. In the future, we will further verify the mechanism of *Bacteroides plebeius* and will further explore the pathogenesis of skeletal muscle atrophy in CKD using a greater clinical sample size.

## AUTHOR CONTRIBUTIONS

**Tingting Pei:** Data curation (equal); methodology (equal); writing – original draft (equal); writing – review and editing (equal). **Daoqi Zhu:** Writing – original draft (equal); writing – review and editing (equal). **Si xia YANG:** Methodology (equal). **Rong Hu:** Methodology (equal). **Fujing Wang:** Methodology (equal). **Jiaxing Zhang:** Data curation (equal). **ShiHua Yan:** Methodology (equal). **Liliang Ju:** Data curation (equal). **zhuoen He:** Data curation (equal). **Zhongxiao Han:** Data curation (equal). **Jinyue He:** Methodology (equal). **Yangtian Yan:** Methodology (equal). **Mingqing Wang:** Conceptualization (equal). **Wei Xiao:** Conceptualization (equal). **YUN MA:** Conceptualization (equal).

## FUNDING INFORMATION

This work was supported by the National Natural Science Foundation of China (81973804, 81673890, 82004336, 81774035 and 82174322), the Natural Science Foundation of Guangdong Province (2017A030313750 and 2022A1515011405) and the Innovation Team of Chronic Kidney Disease with Integrated Traditional Chinese and Western Medicine (2019KCXTD014).

## CONFLICT OF INTEREST

The authors declare that they have no conflict of interest.

## Supporting information


Figure S1.

Table S1.

## Data Availability

The data that support the findings of this study are available from the corresponding author upon reasonable request.
