# Supplementary figures and images for: Bacteroides plebeius improves muscle wasting in chronic kidney disease by modulating the gut‐renal muscle axis
Source: J Cell Mol Med. 2022 Dec 2;26(24):6066–78. doi: 10.1111/jcmm.17626 (PMC9753468; doi:10.1111/jcmm.17626)

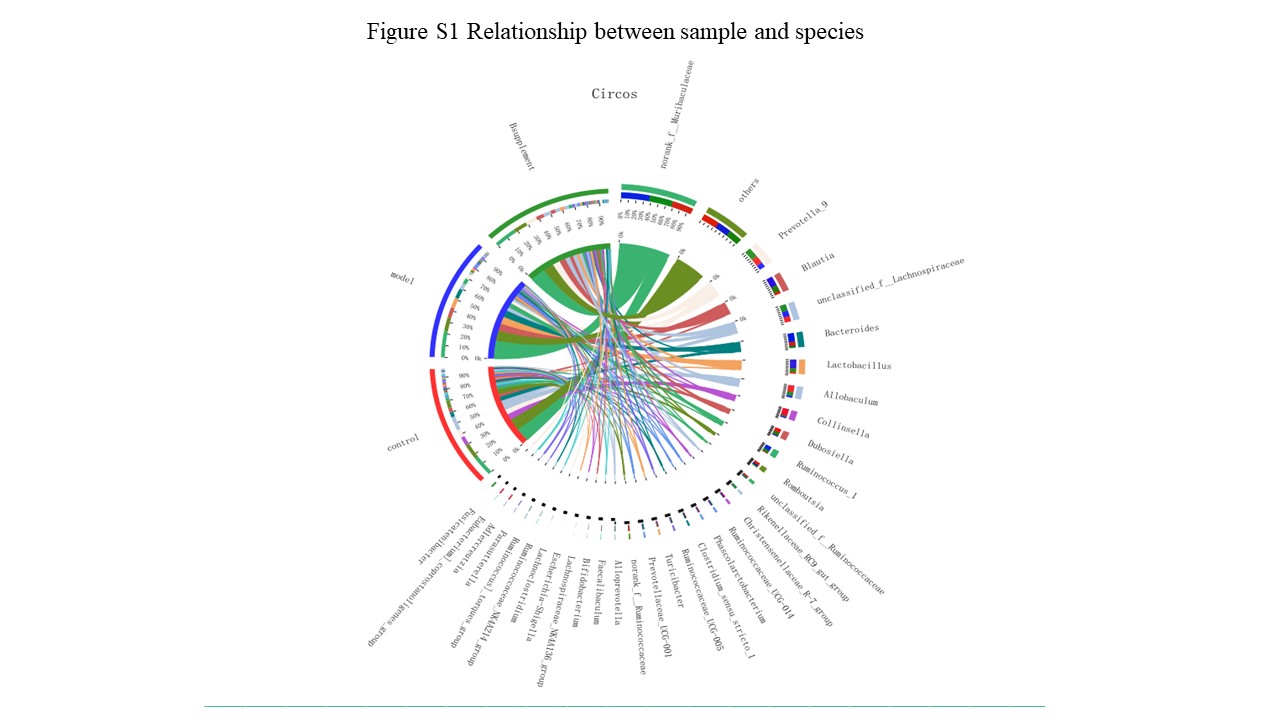

Supplement: Supplementary file 1 — Figure S1. [file JCMM-26-6066-s002.jpg]
